# Stop voicing contrast in American English: Data of individual speakers in trochaic and iambic words in different prosodic structural contexts

**DOI:** 10.1016/j.dib.2018.10.053

**Published:** 2018-10-24

**Authors:** Sahyang Kim, Jiseung Kim, Taehong Cho

**Affiliations:** a Department of English Education, Hongik University, Seoul, Republic of Korea; b Department of Linguistics, University of Michigan, United States; c The Institute for Phonetics and Cognitive Sciences of Language, Department of English Language and Literature, Hanyang University, Seoul, Republic of Korea

## Abstract

The data reported in this article contain eleven (6 female and 5 male) individual speaker’s speech production patterns for the word-initial voiced and voiceless stops (/p,t/ and /b,d/) in American English. The production patterns are documented in the acoustic parameter: the Integrated Voicing Index (IVI) obtained from Voice Onset Time (VOT) and voicing duration in the stop closure (Voicing-in-Closure), in various prosodic contexts: lexically-stressed vs. unstressed; accented (focused) vs. unaccented (unfocused); phrase-initial vs. phrase-medial. The data also contain a CVS file with each speaker׳s mean values of the IVI, VOT and Voicing-in-Closure for each prosodic condition for the voiced and voiceless stops, along with the information about the speaker gender. For further discussion of the data, please refer to the full length article entitled “Prosodic-structural modulation of stop voicing contrast along the VOT continuum in trochaic and iambic words in American English” (Kim et al., 2018).

**Specifications table**

**Table:** 

Subject area	*Linguistics*
More specific subject area	*Phonetics*
Type of data	*Table, figure, spreadsheet*
How data was acquired	*Acoustic measurements based on speech recorded in a laboratory setting*
Data format	*Tables, figures, CVS file*
Experimental factors	*Three main experimental factors were included such as stop voicing (voiced vs. voiceless stops), stress (stressed vs. unstressed conditions in trochaic vs. iambic words), accent (the presence vs. absence of focus-induced pitch accent), and prosodic boundary (the presence vs. absence of Intonational Phrase boundary).*
Experimental features	*Preparation of the data involved acquisition of acoustic data and analyses of voicing reflected in Voice Onset Time and other voicing metrics*.
Data source location	*Hanyang University, Seoul, Korea*
Data accessibility	*Data within the article*
Related research article	*S. Kim, J. Kim, T. Cho, Prosodic-structural modulation of stop voicing contrast along the VOT continuum in trochaic and iambic words in American English, Journal of Phonetics 71 (2018) 65–80*.

**Value of the data**

• The data illustrate eleven individual American English speaker׳s speech patterns for the voiced and voiceless stops in various prosodic contexts, which can be used to understand speaker variation in the phonetic implementation of the phonological stop voicing contrast.
• The data can be used to examine the gender-related difference (six female, five male) in the production of the voiced vs. voiceless stops in American English.
• The attached CVS file contains individual speaker’s mean values for each condition, which can be used to run additional statistical analyses.
• The data with respect to individual speaker׳s production of the voiced and voiceless stops in various prosodic contexts will inform further studies of individual speech variation under the rubric of the phonetics-prosody interface.
• The data will foster further research on cross-linguistic aspects of speech production in reference to higher-order linguistic structures as exemplified in Cho et al. [2].

## 1 Data

The data presented in this article illustrate eleven American English speaker’s individual patterns of the acoustic phonetic realization of the voiced and voiceless stop consonants (/p/*-*/b/; /t/*-*/d/) in various prosodic contexts, which is related to Kim et al. [1].

### 1.1 Initial stops in the trochaic words: in the stressed condition

**Fig. 1:**
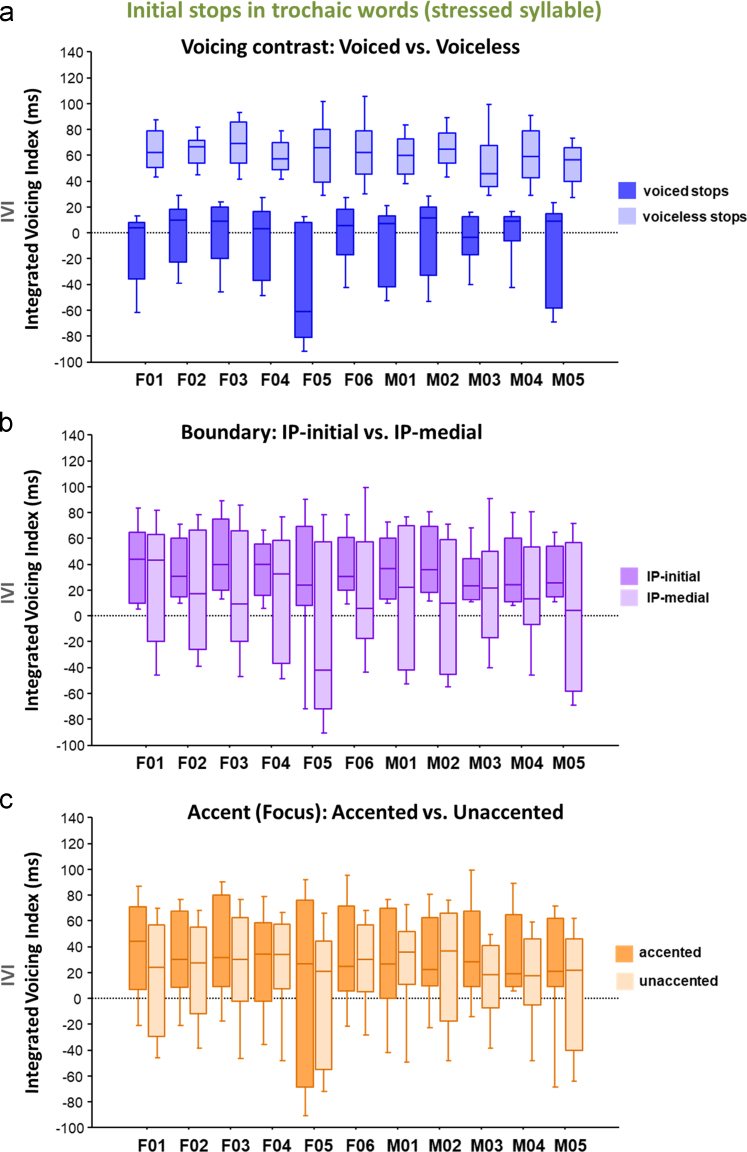
Boxplots for the distribution of the Integrated Voicing Index (IVI) for the initial stops in the *stressed* syllable across 11 speakers: (a) the difference in the IVI between the voiced and voiceless stops; (b) the difference in the IVI between the IP-initial and IP-medial positions; and (c) the difference in the IVI between the accented (focused) and unaccented (unforced) conditions. The IVI was defined as a combined sum of VOT (as a positive value) and Voicing-in-Closure (as a negative value).

Fig. 1 illustrates how the eleven individual speakers of American English produced voiceless vs. voiced stops in the initial stressed syllables in trochaic words. This provides the information of how the phonological voicing contrast in the stressed initial syllable is phonetically implemented along the phonetic voicing dimension: the IVI, the Integrated Voicing Index which was obtained from VOT and Voicing-in-Closure (Fig. 1a) (see below for more information about the IVI). Fig. 1 also illustrates how the individual speakers produced the stops (the voiceless and voiced stops combined) in various prosodic contexts: in the two prosodic boundary conditions, IP-initial vs. IP-medial position (Fig. 1b) and in the two prominence conditions, accented (focused) vs. unaccented (unfocused) (Fig. 1c). The horizontal axes in the figure refer to speaker ID number along with the gender information.

**Fig. 2:**
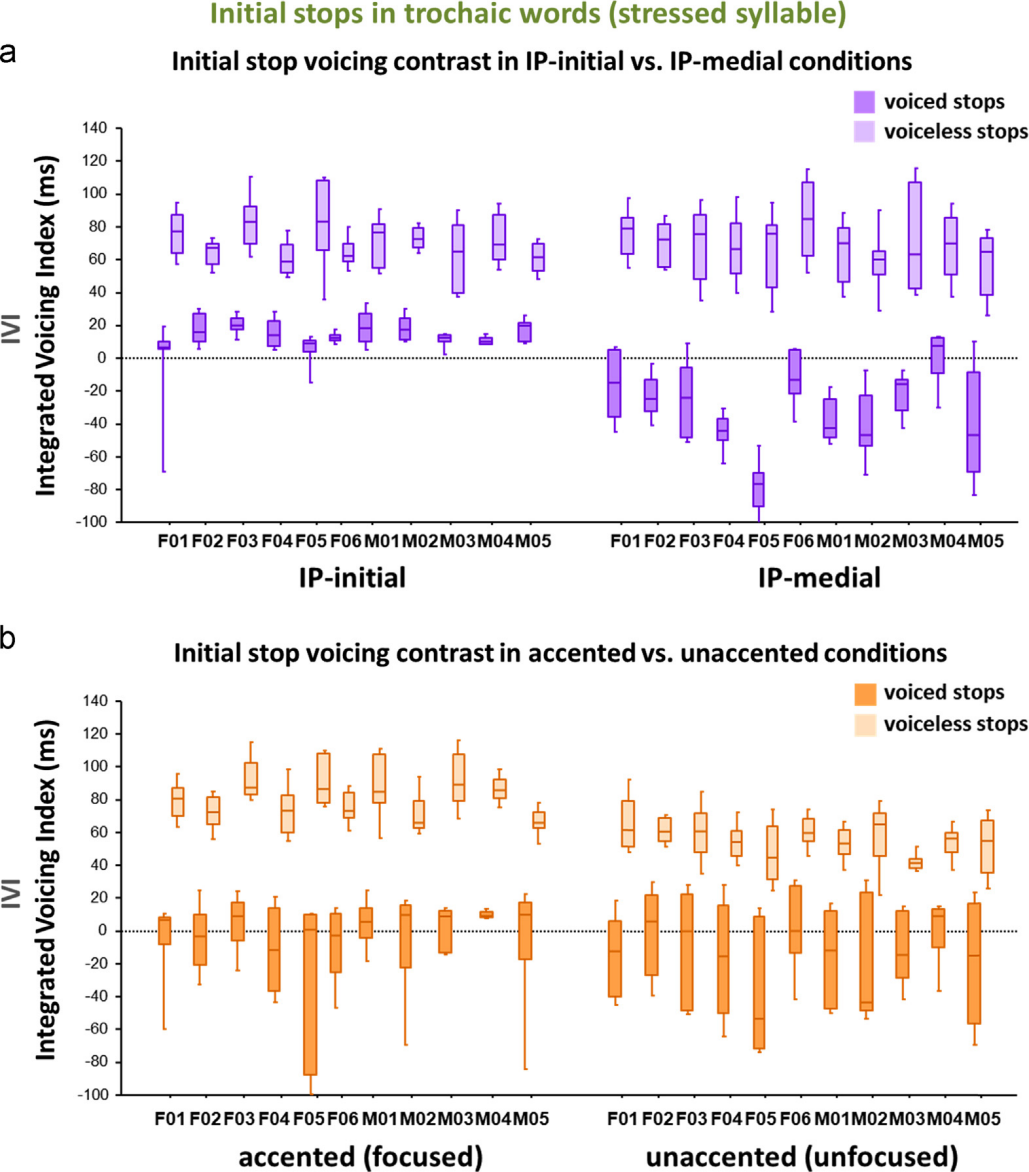
Boxplots for the distribution of the Integrated Voicing Index (IVI) of the initial stop voicing contrast (the voiceless vs. voiced stops) in the stressed syllable across 11 speakers as a function of (a) the boundary conditions (IP-initial vs. IP-medial) and (b) the prominence conditions (accented/focused vs. unaccented/unfocused). The IVI was defined as a combined sum of VOT (as a positive value) and Voicing-in-Closure (as a negative value).

Fig. 2 illustrates how individual speakers produced the voiceless vs. voiced stops (i.e., the voicing contrast) as a function of two prosodic factors: Boundary (IP-initial vs. IP-medial) (Fig. 2a) and Prominence (accented vs. unaccented) (Fig. 2b).

### 1.2 Initial stops in the iambic words: in the unstressed condition

**Fig. 3:**
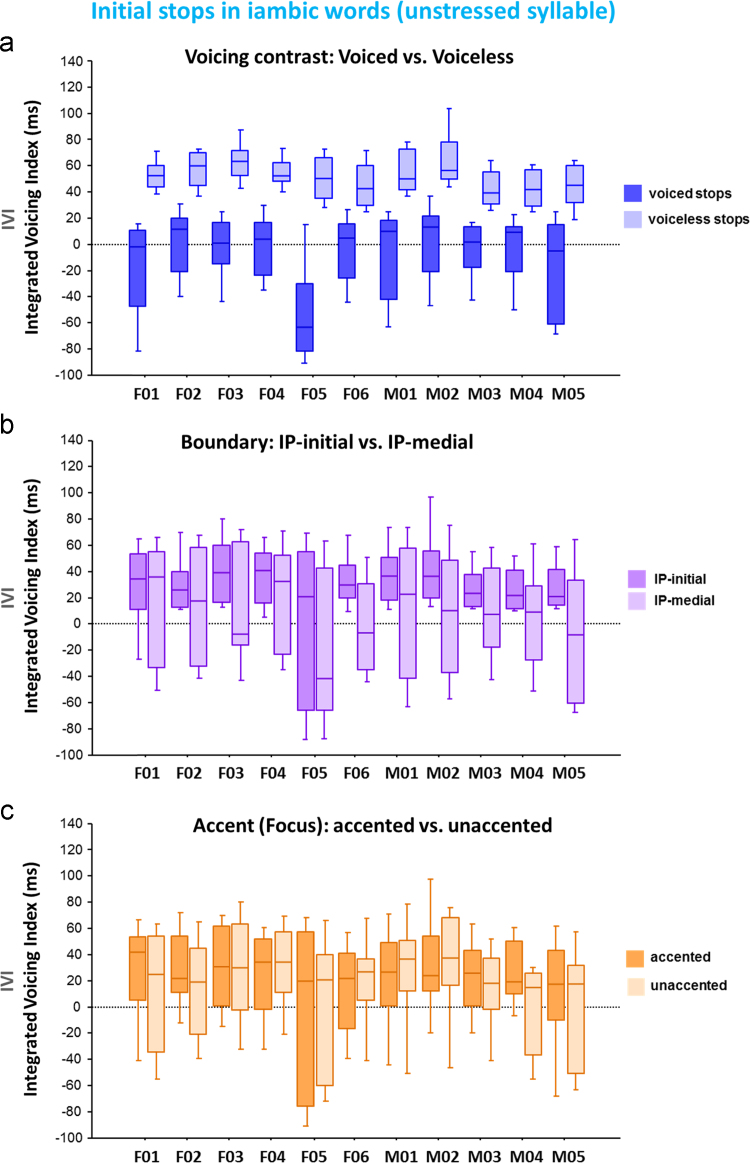
Boxplots for the distribution of the Integrated Voicing Index for the initial stops in the unstressed syllable across 11 speakers: (a) the difference in the IVI between the voiced and voiceless stops; (b) the difference in the IVI between the IP-initial and IP-medial positions; and (c) the difference in the IVI between the accented (focused) and unaccented (unforced) conditions. The IVI was defined as a combined sum of VOT (as a positive value) and Voicing-in-Closure (as a negative value).

Fig. 3 illustrates how the eleven individual speakers produced the voiceless vs. voiced stops in the initial unstressed syllables in iambic words in American English. This provides the information of how the phonological voicing contrast in the unstressed initial syllable is phonetically implemented along the phonetic voicing dimension: IVI, the Integrated Voicing Index (Fig. 3a). Fig. 3 also illustrates how the individual speakers produced the stops (the voiceless and voiced stops combined) in various prosodic contexts: in the two prosodic boundary conditions, IP-initial vs. IP-medial position (Fig. 3b) and in the two prominence conditions, accented (focused) vs. unaccented (unfocused) (Fig. 3c). The horizontal axes in the figure refer to speaker ID number along with the gender information.

**Fig. 4:**
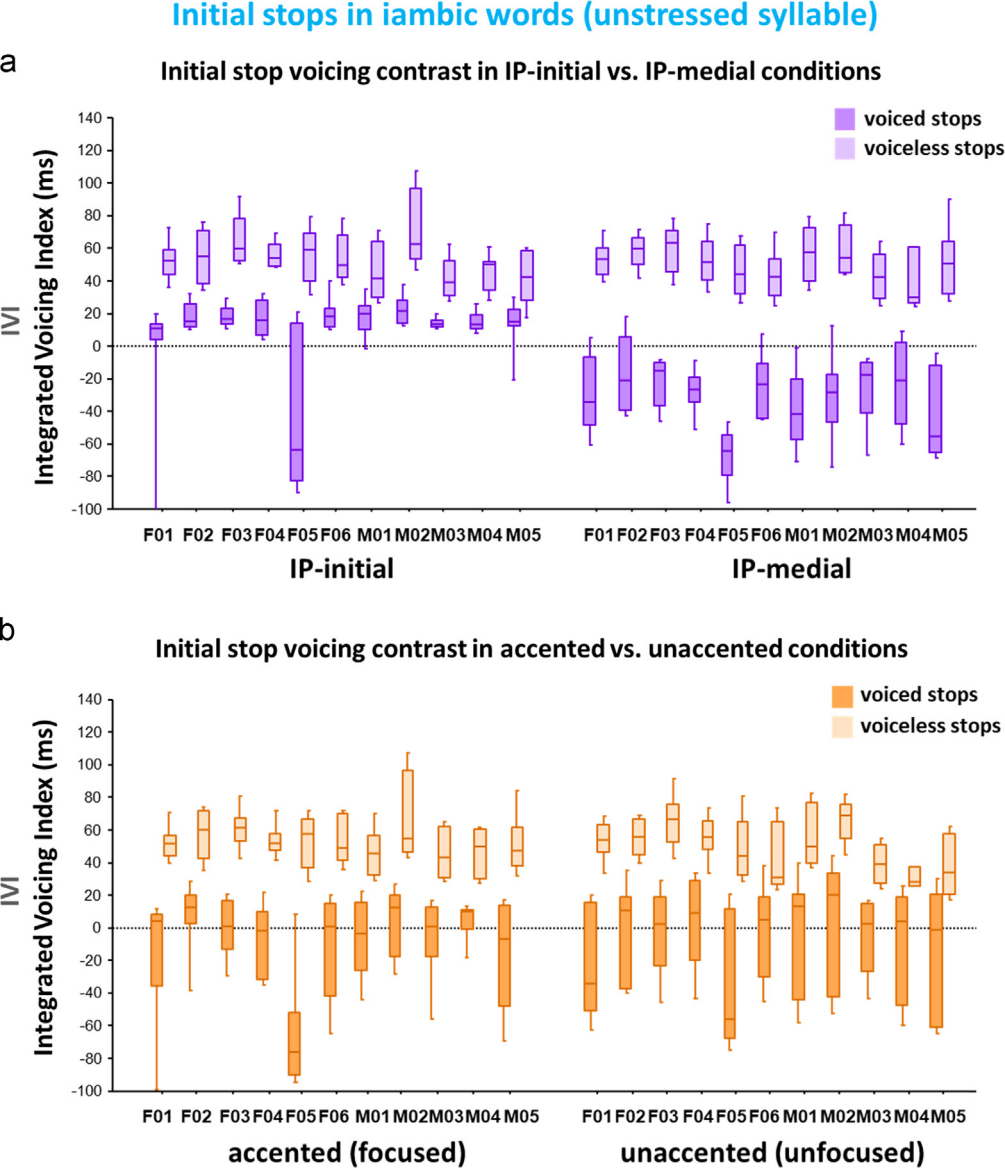
Boxplots for the distribution of the Integrated Voicing Index (IVI) of the initial stop voicing contrast (the voiceless vs. voiced stops) in the unstressed syllable across 11 speakers as a function of (a) the boundary conditions (IP-initial vs. IP-medial) and (b) the prominence conditions (accented/focused vs. unaccented/unfocused). The IVI was defined as a combined sum of VOT (as a positive value) and Voicing-in-Closure (as a negative value).

Fig. 4 illustrates how the individual speakers produced the voiceless vs. voiced stops (i.e., the voicing contrast) as a function of two prosodic factors: Boundary (IP-initial vs. IP-medial) (Fig. 4a) and Prominence (accented vs. unaccented) (Fig. 4b).

### 1.3 Individual speakers’ mean values for each condition

The CVS file attached to this article contains mean values of the IVI, VOT, and Voicing-in-Closure.

The organization of the file in terms of experimental factors is illustrated in Table 1. As shown in the table, each speaker is labeled with ‘F’ (female) or ‘M’ (male), and four experimental factors: Stress (stressed vs. unstressed), Boundary (IP-initial vs. IP-medial), Accent (accented/focused vs. unaccented/unfocused), Voicing (voiced vs. voiceless). This file can be used for carrying out further analyses of the data, and compared to previous data on the talker-specific realization of VOT (e.g., [6]; cf. [7]).

**Table 1:** Part of the CVS file that illustrates the organization of the file with respect to experimental conditions. The file contains the mean value of each condition for IVI, VOT and Voicing-in-Closure.

Speaker ID	Stress	Boundary	Accent	Voicing	Mean IVI (ms)	Mean VOT(ms)	Mean Voicing-in-Closure (ms)
F01	Stressed (trochaic)	IP-initial	Accented (focused)	Voiced	−12.4	7.4	19.8
				Voiceless	78.3	78.3	0.0
			Unaccented (unfocused)	Voiced	11.7	11.7	0.0
				Voiceless	71.5	71.5	0.0
		IP-medial	accented (focused)	Voiced	−2.8	8.6	11.4
				Voiceless	82.0	82.0	3.6
			Unaccented (unfocused)	Voiced	−30.3	4.7	34.9
				Voiceless	59.5	59.5	6.5
	Unstressed (iambic)	IP-initial	Accented (focused)	Voiced	−22.4	6.3	28.6
				Voiceless	48.8	48.8	0.0
			Unaccented (unfocused)	Voiced	17.4	17.4	0.0
				Voiceless	60.2	60.2	0.0
		IP-medial	Accented (focused)	Voiced	−13.9	8.4	22.3
				Voiceless	56.3	56.3	5.0
			Unaccented (unfocused)	Voiced	−47.1	0.0	47.1
				Voiceless	46.3	46.3	7.4

## 2 Experimental design, materials and methods

### 2.1 Participants

Six female and five male American English speakers in their 20s and early 30s were paid to participate in the acoustic recording (See [1] for more information about the speakers).

### 2.2 Speech materials for acoustic recordings

As shown in Table 2, eight words were used as test words. Half of the words were stress-initial words (trochaic) and the other half were stress-final (iambic), so that the first syllables were stressed vs. unstressed (See [1] for more information about the speech materials).

**Table 2:** List of target words.

	Initial stop: voiceless	Initial stop: voiced
Trochaic words (stressed initial)	*pánel, tánner*	*bánner, Dániel*
Lambic words (unstressed initial)	*panáche, Teníse*	*banál, Deníse*

The test words were produced in carrier sentences in four critical conditions as exemplified in Table 2. Each carrier sentence consisted of a background sentence followed by the target-bearing test sentence. The first sentence helped the speaker to produce the target-bearing sentence with intended prosodic conditions. In Table 3a and c, the test word (*bánner*) in the second sentence is contrastive with a corresponding word (e.g., *pánel*) in the first sentence. The contrastive focus was meant to induce the speaker to place a nuclear pitch accent on the test word in the accented condition. For the unaccented condition as in Table 3b and d, a contrastive focus fell elsewhere in the sentence, so that the test word became unaccented. As for the boundary conditions, to induce an Intonational Phrase (IP) boundary before the test word (IP-initial condition), the test word was aligned with a major syntactic boundary between a subordinate clause and a main clause as in Table 3a and b (e.g., *But after JOHN says ‘banana’, ‘BANNER again’ will be the next phrase to say*.).To induce a Word boundary before the test word (IP-medial condition), the two-word sequence formed part of a single object noun phrase within the same syntactic phrase, as in Table 3c and d (e.g., *To say “banana banner again” with me*…).

**Table 3:** The test word *banner* produced in carrier sentences in four critical conditions: two boundary conditions (IP-initial, IP-medial) x two accent conditions (accented, unaccented). The accented words are marked in bold, and the test word is underlined.

**a. IP-initial, Accented (where ‘*****#*****’=IP boundary)**
*After I say ‘banana,’ ‘**PANEL** again’ will be the next phrase to say.*
*But after JOHN says ‘banana,’# ‘BANNER again’ will be the next phrase to say.*
**b. IP-initial, Unaccented (where ‘*****#*****’=IP boundary)**
*After **I** say ‘banana,’ ‘banner again’ will be the **NEXT** phrase to say.*
*But after **JOHN** says ‘banana,’**#** ‘banner again’ will be the **FINAL** phrase to say.*
**c. IP-medial, Accented (where ‘*****#*****’=Word boundary in phrase-medial position)**
*To say ‘banana **PANEL** again’ with me is going to be difficult.*
*But to say ‘banana**#BANNER** again’ with me is going to be easy.*
**d. IP-medial, Unaccented (where ‘*****#*****’=Word boundary in phrase-medial position)**
*To say ‘banana banner again’ with **JOHN** is going to be difficult.*
*But to say ‘banana#banner again’ with **ME** is going to be easy.*

The recordings took place in a sound-attenuated booth at the Hanyang Phonetics and Psycholinguistics Lab at a sampling rate of 44 kHz using a SHURE KSM 44 condender microphone and a Tascam HD-P2 digital recorder. Sentences were presented on a computer screen in a randomized order and repeated four times across four blocks. Speakers were introduced to read the carrier sentences aloud with the meaning contrast in mind. At the time of recording, when the experimenter, a trained prosody transcriber, noticed any production error, she asked the speaker to read the sentence a few more times to obtain as natural utterances as possible. The recording session ran for about 60 min with two 5 min breaks. A total of 1408 tokens were obtained: 2 boundary conditions (IP-initial, IP-medial) × 2 accent conditions (accented, unaccented) × 8 target words × 4 repetitions × 11 speakers. Two trained phoneticians crosschecked the recorded data in terms of prosodic conditions. When the cross-checkers disagreed on any intended prosodic condition in a target-bearing sentence, that token was excluded from further analyses. This crosschecking procedure excluded 358 tokens, leaving 1050 tokens for the data analyses.

### 2.3 Measurements

The following acoustic duration measures were taken from the initial syllable of each target word, using Praat [3].

#### 2.3.1 (Positive) VOT (voicing lag)

Positive VOTs of both voiceless and voiced stops were measured from the stop release to the onset of voicing (the first regular waveform) for the following vowel. VOT included any observable voicing lag, even when a voiced stop was produced with voicing (phonation) during the closure. The measurement procedure is largely in line with Abramson and Whalen [4] and Davidson [5].

#### 2.3.2 Voicing-in-Closure

The voiced interval during the stop closure was measured for both the voiced and voiceless stops, as indicated by voicing bars in the spectrogram in consultation with waveforms. Voicing-in-Closure included any continued voicing murmur (with two or more clear voicing bars on the spectrogram) during the closure (between the F2 offset of the preceding vowel and the stop release) as well as any voicing lead before the burst of the voiced stop. (This measure can be taken to be negative VOT if it is defined to include any portion of prevoicing during the closure whether intermittent or continuous).

#### 2.3.3 Integrated Voicing Index (IVI)

The IVI was defined as a combined sum of VOT (as a positive value) and Voicing-in-Closure (as a negative value). This voicing index was devised to weigh the relative contribution of VOT and Voicing-in-Closure to the voicing contrast, allowing us to assess the phonetic voicing of both voiceless and voiced stops along a single integrated dimension of the phonetic voicing (See Kim et al. [1] for further discussion of the usefulness of this metric).
