# Supplementary material for: Stop voicing contrast in American English: Data of individual speakers in trochaic and iambic words in different prosodic structural contexts
Source: Data Brief. 2018 Oct 24;21:980–8. doi: 10.1016/j.dib.2018.10.053 (PMC6222083; doi:10.1016/j.dib.2018.10.053)
Supplement: Supplementary file 1 — Supplementary material. [file mmc1.docx]

**Conflict of Interest Statement**

**Title: Stop voicing contrast in American English: Data of individual speakers in trochaic and iambic words in different prosodic structural contexts**

**Authors: Sahyang Kim, Jiseung Kim and Taehong Cho**

**We hereby state that any potential conflicts of interest do not exit with respect to this submission. We followed the ethical guidelines as set by our Institute (Hanyang University), and we disclosed all the financial support information.**

**Taehong Cho**

**(Also on behalf of co-authors, Jiseung Kim and Sahyang Kim).**
